# Depression among the general adult population in Jiangsu Province of China: prevalence, associated factors and impacts

**DOI:** 10.1007/s00127-018-1568-0

**Published:** 2018-07-30

**Authors:** Shurong Lu, Nicola Reavley, Jinyi Zhou, Jian Su, Xiaoqun Pan, Quanyong Xiang, Yongqing Zhang, Brian Oldenburg, Ming Wu

**Affiliations:** 1 Department of Chronic Disease Control, Jiangsu Provincial Centre for Disease Control and Prevention, 172 Jiangsu Road, Nanjing, 210009 Jiangsu China; 2 Centre for Mental Health, Melbourne School of Population and Global Health, University of Melbourne, Melbourne, VIC Australia; 3 Noncommunicable Disease Unit, Melbourne School of Population and Global Health, University of Melbourne, Melbourne, VIC Australia

**Keywords:** Depression, Prevalence, Associated factor, The Patient Health Questionnaire (PHQ-9), China

## Abstract

**Purpose:**

This study aimed to assess the prevalence of depression and to investigate its associated factors and impacts on daily life functioning among the general adult population in Jiangsu Province of China.

**Methods:**

As part of the *Jiangsu Provincial Survey on Chronic Disease and Behavioural Risk Factors* (*2010*), a sample of 8400 community residents aged ≥ 18 years were recruited. Study data were collected through a questionnaire-administered face-to-face interview. Depression was measured by the Patient Health Questionnaire.

**Results:**

The estimated prevalence of depression was 0.56% (0.54% in men and 0.58% in women). Increased risk of depression was found to be associated with rural residents (OR 2.24, 95% CI 1.33–3.78) and the comorbidity of chronic diseases (OR 3.83, 95% CI 1.33–11.02). Respondents with depression reported an average of 11.75 unhealthy days caused by physical illnesses and 8.31 unhealthy days by mood problems within the previous 30 days. Depression was also found to be related to worse self-ranked health status, worse relationships with families and lower life satisfaction.

**Conclusions:**

A low prevalence of depression was found in this population of China, though it is not clear the extent to which it reflects issues related to the measurement and/or other factors of the survey. Depression was found to be related to poorer health and poorer life functioning. Further research into the link between depression and access to mental health services in rural areas is necessary. Meanwhile, depression among chronic disease patients should be addressed in clinical settings, health plans and resources allocation.

## Introduction

Depression is a common mental illness, with an estimated 300 million people affected globally [1]. It significantly impacts on quality of life, health and relationships, thereby contributing to poorer functioning at work, school and within the family [2]. In 2015, depression was ranked by the World Health Organisation as the single largest contributor to global disability, accounting for 7.5% of all years lived with disability [3]. Due to population growth, urbanisation and changing age structures, prevalent cases of depressive disorders and their related burden of disease are on the rise globally [1, 4].

Reliable estimates of the prevalence of depression and its risk factors are needed to estimate disease burden, inform health-care policy and assist in the development of preventive strategies. There are large differences in the prevalence of depression across countries and regions [5]. Generally, it is believed that countries within East Asia, including China, have lower prevalence of common mental disorders than other regions of the world, whereas English-speaking countries have the highest lifetime prevalence rates [6]. However, it is not clear the extent to which this reflects cultural/ethnic differences and/or issues related to measurement.

There is some evidence to suggest that the prevalent cases of depression among the general population in China may have risen in recent years due to rapid socioeconomic changes, such as aging, widening urban–rural gap in economic status and increasing rates of divorce [7]. The pace and spread of chronic diseases may also contribute to such an increase, as a growing number of studies suggest that the presence of co-morbid depression and depressive symptoms is higher among people with chronic diseases (e.g. diabetes, stroke and cardiac disease), compared to the general population [8, 9]. Evidence also suggests that depression and chronic diseases may share common risk factors, including obesity-promoting behaviours (e.g. physical inactivity, hypercaloric diets) [10] and activation of the neuroendocrine and inflammatory responses (resulting in increased cortisol, catecholamines and cytokines) [11].

Over the past few decades, some national and regional mental health surveys have been conducted in China and have reported on the prevalence of depression. The results varied considerably across studies [12]. For example, Gu et al. [13] reported in a systematic review that the 2-week prevalence rate of major depressive disorder ranged from 0.3 to 3.1%. Most previous studies in China employed *the Composite International Diagnostic Interview* (*CIDI*) and/or *the Structured Clinical Interview for DSM* (*SCID*) [12], which are believed to be the “gold standard” tools for the assessment of mental disorders. However, these diagnostic instruments are quite lengthy to administer and, therefore, place a considerable burden on interviewers and respondents. Implementation can therefore be difficult in countries like China [14].

One of the most widely used shorter screening tools for the presence of depressive symptoms is the Patient Health Questionnaire (PHQ-9). It is valued for its brevity and tight match to the diagnostic criteria outlined in *the Diagnostic and Statistical Manual Disorders (DSM-IV)* [15]. Globally, this tool has been used in a variety of settings such as primary care, specialist medical services and in the community [16, 17]. Its validity and reliability have been shown in different cultural contexts as well [18–20]. A Chinese version of the PHQ-9 has been developed and validated in several studies. It was found to have acceptable psychometric properties among primary care patients [21] and community residents [22]. A small number of studies have also used this tool among some important population subgroups including elderly people and clinical patients in Mainland China [23], but no such studies have been conducted in the general population. Therefore, the aim of this study was to use the PHQ-9 to estimate the prevalence of depression among the general adult population in the Jiangsu Province of China. Further aims were to explore its associated factors and impacts on daily life functioning.

## Methods and materials

### Settings, participants and data collection

A population-based cross-sectional survey on chronic disease and behavioural risk factors was conducted in the Jiangsu Province of China during 2010–2011, under the approval of the *Jiangsu Provincial Department of Health* (the present *Jiangsu Provincial Commission of Health and Family Planning*). With a population of 80 million, Jiangsu is one of the areas of China undergoing most rapid socioeconomic development. Jiangsu comprises three regions: the south, the middle and the north, with great variations in climate, culture and lifestyle across regions. According to official statistics [24], in 2010, the per capita gross domestic product (GDP) for the south, middle and north region was 11,744, 7005 and 4398 USD, respectively, demonstrating a significant gradient.

This survey adopted a multi-stage, randomly stratified cluster sampling scheme to select participants [25]. In the first stage, 14 counties/districts, representative of Jiangsu Province in terms of geographical distribution, economic development and population composition, were selected as the primary sampling units. In the second stage, four towns/streets were selected from each county/district using the proportional to population size sampling method. Thirdly, three villages/communities were selected from each selected town/street. Within selected villages/communities, 50 households (private dwellings) were randomly sampled, and one eligible resident (aged ≥ 18 years, residing in the household for a minimum of 6 months prior to the survey) was selected by the Kish grid method [26] to participate in the survey from all eligible people within each household. The detailed sampling procedure has been documented elsewhere [27]. A total of 8400 individuals were selected. The data presented in this article were collected as part of this survey.

Data were collected using a standard questionnaire-administered face-to-face interview by well-trained public health practitioners. Questionnaire items covered demographic characteristics, lifestyle factors and comorbidities of most common chronic diseases among Chinese [28]. Information on self-rated health status, relationships with families and satisfaction with life was also collected. All interviews were conducted in separated rooms for the sake of privacy. Prior to each interview, the participant was informed of the survey procedure and the confidentiality of information they provided. All participants gave their written informed consent and the survey was approved by the *Ethical Committee of Jiangsu Provincial Centre for Disease Control and Prevention*.

### Socio-demographic characteristics

Residence and region of respondents were categorised according to the government administrative divisions, while marital status was based on self-report. For the education level, illiterate to finishing primary school was defined as “low”, finishing middle school or equivalent as “medium” and finishing university or equivalent as “high”. The household income level was classified by the tertiles of annual household income per capita in the previous year of the survey, and those below the first tertile, between the first and second tertile and above the second tertile were classified as “low”, “medium” and “high”, respectively.

### Measures

#### Depression and daily life functioning

Depression and its severity were measured by the PHQ-9, which consists of nine items, each describing a typical depressive symptom. Response categories, based on frequency of a particular symptom over 2 weeks prior to the interview were scored 0, 1, 2 and 3, for “Not at all”, “Several days”, “More than half the days”, and “Nearly every day”, respectively. Thus, the whole questionnaire yields a severity score from 0 to 27. The tenth item of PHQ-9 is about the impact of the above symptoms on life performance (“*How difficult have those problems made it for you to do your work, take care of things at home, or get along with other people?*”), with the following response categories: “Not difficult at all”, “Somewhat difficult”, “Very difficult” and “Extremely difficult”. Based on the findings of the pilot study, we further adapted the questionnaire by including two additional options, “*Don’t know*” and “*Refuse to respond*”, to each of the items in the questionnaire to make sure that all respondents would find their most suitable options.

Consistent with international use of the PHQ [15, 18, 19], if a person endorsed the tenth item of the PHQ-9 (“*How difficult have those problems made it for you to do your work, take care of things at home, or get along with other people?*”) as “Somewhat difficult”, “Very difficult” or “Extremely difficult”, and he/she achieved a PHQ score ≥ 5, then this person was defined as having “depression”. Individuals with a PHQ score ≥ 10 were defined as having “major depression”. The severity of depression was classified by PHQ score as the following: score 0–4 for *none*, 5–9 for *mild*, 10–14 for *moderate*, 15–19 for *severe* and 20–27 for *extremely severe* [15].

To further examine the impacts of depression on daily life functioning, the following three questions were asked: ① *How do you describe your current health status?* ② *How would you describe your relationship with your families in the past 12 months?* ③ *Overall, how satisfied were you with life in the past 12 months?* Questions ① and ② were followed with responses of “Very good”, “Good”, “Fair”, “Bad” and “Very bad”, while question ③ was followed with responses of “Very satisfied”, “Satisfied”, “Fair”, “Unsatisfied” and “Very unsatisfied”. Participants were also asked to give the number of days that they felt unhealthy due to physical illnesses or mood problems in the previous 30 days.

#### Presence of chronic disease

There were seven common chronic diseases included in this study, including hypertension, diabetes, cancer, stroke, myocardial infarction, chronic obstructive pulmonary disease and asthma. Hypertension and diabetes were defined by both self-report (“*Have you ever been diagnosed with hypertension/diabetes in a community or above level hospital?*”) and objective measurements/tests according to the corresponding criteria of guidelines of Chinese, whereas the other five were based on self-report [25].

### Statistical analysis

In total, 8400 individuals completed all the assessments (response rate = 100%). Given the influence on completeness of the PHQ-9 scoring, 101 individuals were excluded from the analysis because of choosing “Don’t know” or “Refuse to respond” in at least one item of the PHQ-9. Therefore, 8299 cases were included for the analysis. Compared with the analysed group, there were higher percentages of women, people aged ≥ 65 years, individuals with low education, low income, comorbidity of chronic disease and people who were divorced/widowed/separated in the excluded group. We further compared the study sample with census statistics of the Jiangsu population and found that they were similar in most socio-demographic characteristics such as residence, regional distribution and education level, but the sample had a lower percentage of men (45.43 vs. 50.38%, *P* < 0.001) and people aged 34 years or younger (12.11 vs. 32.60%, *P* = 0.007) than the general population.

Because of the difference in gender and age between the sample and the population, we weighted the data by gender and age according to the latest census data of the Jiangsu population (2010), when estimating the prevalence, the distribution of PHQ scores and impacts of depression on daily life functioning. The *χ*^2^ test was used to explore the differences in sample characteristics, as well as to measure univariate associations between depression and analysed categorizing variables. Multivariate logistic regression was used to identify the independent risk factors of depression, with odds ratios (ORs) and the corresponding 95% confidence intervals (CIs) being calculated. Spearman correlation analysis was conducted to measure the quantitative association between PHQ score and unhealthy days caused by physical illness or mood problems. All statistical analyses were performed using *SPSS* software (IBM SPSS Statistics 24.Ink), and *P* values < 0.05 (two sided) were considered statistically significant.

## Results

### Demographic characteristics of participants

The study population (men, 45.43%) was aged between 18.02 and 94.93 years, with a mean age of 52.37 (SD 14.81) years. The demographic characteristics of participants are demonstrated in Table 1. Young people (aged 34 years or less) accounted for 12.11% of the sample and the elderly (aged 65 years or above) accounted for 20.17%. There were 42.88% participants from rural areas, and people from the region of south, middle and north accounted for about one-third of the sample, respectively. There were 47.67% of all respondents with “Low” education and 38.15% with “Low” household income. As this study was conducted among the adult population, more than 80% of the sample were married, 3.83% were single and 11.37% divorced/widowed/separated. Noticeably, 43.35% of the participants had one kind of chronic disease (i.e. cancer, stroke, myocardial infarction, hypertension, diabetes, chronic obstructive pulmonary disease or asthma), and nearly another 10% had two or more comorbid chronic diseases (Table 1).

**Table 1:** Demographic characteristics of participants

	*N*	%
*Age group (years)*		
18–34	1005	12.11
35–44	1714	20.65
45–54	1896	22.85
55–64	2010	24.22
≥ 65	1674	20.17
*Residence*		
Urban	4740	57.12
Rural	3559	42.88
*Region*		
South	2964	35.72
Middle	2378	28.65
North	2957	35.63
*Education*		
Low	3956	47.67
Medium	3827	46.11
High	516	6.22
*Household income level*		
Low	2863	38.15
Medium	2549	33.97
High	2092	27.88
*Marital status*		
Married	7037	84.79
Single	318	3.83
Divorced/widowed/separated	944	11.37
*Number of comorbid NCDs* ^a^		
0	3890	46.90
1	3596	43.35
2	676	8.15
≥ 3	133	1.60
*Total*	8299	100

^a^Non-communicable chronic diseases, including cancer, stroke, myocardial infarction, hypertension, diabetes, chronic obstructive pulmonary disease and asthma

### Prevalence estimates of depression and major depression

As shown in Table 2, the 2-week prevalence of depression among the general adult population in the Jiangsu Province was 0.56% (0.54% in men and 0.58% in women, *P* = 0.418), while that of major depression was 0.22%. Both the prevalence of overall depression and major depression increased with age (both *P* values < 0.001), with people aged 65 years or older having higher prevalence estimates (1.52% of depression and 0.87% of major depression, respectively) than other age groups. The prevalence of depression among rural residents was more than double that of urban residents (0.86 vs. 0.36%, *P* = 0.001). Similarly, residents from the north or the middle region of Jiangsu had a higher prevalence of depression than their southern counterparts (0.77 vs. 0.72 vs. 0.22%, *P* = 0.002). While there was a similar trend in the prevalence of major depression, the difference was not statistically significant.

**Table 2:** Estimated prevalence of depression and major depression among the general adult population in Jiangsu, China (*N* = 8299)

	Depression		Major depression	
	Mean ± SE, %	*P* value	Mean ± SE, %	*P* value
*Age group (years)*				
18–34	0.16 ± 0.14	< 0.001	0.08 ± 0.10	< 0.001
35–44	0.33 ± 0.14		0.05 ± 0.06	
45–54	0.48 ± 0.16		0.15 ± 0.09	
55–64	0.94 ± 0.22		0.25 ± 0.11	
≥ 65	1.52 ± 0.30		0.87 ± 0.23	
*Residence*				
Urban	0.36 ± 0.10	0.001	0.16 ± 0.07	0.083
Rural	0.86 ± 0.18		0.32 ± 0.11	
*Region*				
South	0.22 ± 0.10	0.002	0.10 ± 0.07	0.114
Middle	0.72 ± 0.20		0.30 ± 0.13	
North	0.77 ± 0.18		0.28 ± 0.11	
*Education*				
Low	0.99 ± 0.17	0.001	0.40 ± 0.11	0.040
Medium	0.38 ± 0.11		0.14 ± 0.07	
High	–		–	
*Household income level*				
Low	0.98 ± 0.21	< 0.001	0.27 ± 0.12	0.046
Medium	0.22 ± 0.11		0.06 ± 0.06	
High	0.48 ± 0.17		0.36 ± 0.14	
*Marital status*				
Married	0.54 ± 0.10	0.005	0.20 ± 0.06	0.004
Single	–		–	
Divorced/widowed/separated	1.42 ± 0.41		0.79 ± 0.30	
*Number of comorbid NCDs* ^a^				
0	0.38 ± 0.11	< 0.001	0.14 ± 0.07	< 0.001
1	0.62 ± 0.14		0.20 ± 0.08	
2	1.39 ± 0.49		0.76 ± 0.36	
≥ 3	3.52 ± 1.66		2.06 ± 1.29	
*Total*	0.56 ± 0.09		0.22 ± 0.06	

Estimates with SE > mean were not shown for unreliability

^a^Non-communicable chronic diseases, including cancer, stroke, myocardial infarction, hypertension, diabetes, chronic obstructive pulmonary disease and asthma

As the number of people with “High” level of education or “Single” marital status among the sample was relatively small (516 and 318, respectively), no reliable prevalence estimates for these groups could be calculated. Available data showed that depression was more prevalent among individuals with low education (0.99%), low income (0.98%) or among people who were divorced/widowed/separated (1.42%). The prevalence of depression and major depression also increased with the number of comorbid chronic diseases (both *P* < 0.001). For respondents with three or more comorbidities, the prevalence of depression and major depression was sixfold (3.52 vs. 0.56%) and ninefold (2.06 vs. 0.22%) higher than that among the general population (Table 2).

The study population had a mean PHQ score of 0.90 (95% CI 0.83–0.96). Women had higher scores than men (0.98 vs. 0.81, *P* < 0.001) and rural residents had higher scores than urban ones (1.06 vs. 0.79, *P* < 0.001). Individuals who scored ≥ 10 accounted for 0.92% (95% CI 0.68–1.16%) of all participants (0.80% of men and 1.03% of women, *P* = 0.389). The proportions of the severity of depression were also calculated. Levels of “none (scored 0–4)”, “mild (scored 5–9)”, “moderate (scored 10–14)”, “severe (scored 15–19)” and “extremely severe (scored 20–27)” accounted for 93.75, 5.11, 0.84, 0.22 and 0.08%, respectively (data not shown).

### Factors associated with depression

Table 3 shows the association between depression and socio-demographic factors and comorbid chronic diseases by gender. Multivariate logistic regression analyses showed that women had a higher risk of depression compared with men, though the difference was not statistically significant (OR 1.24, 95% CI 0.75–2.07). The risk of depression dramatically increased with age (*P* for trend < 0.001). Meanwhile, the risk of depression among rural residents was more than double that of urban ones (OR 2.24, 95% CI 1.33–3.78), consistently among both men (OR 2.39, 95% CI 1.05–5.45) and women (OR 2.17, 95% CI 1.10–4.28). Data also showed that the risk of depression increased with the number of comorbid chronic diseases (OR 3.83 for the “≥ 3” group, 95% CI 1.33–11.02). By gender, women in the middle and north regions had a much higher risk of depression than those living in the south (ORs were 4.47 for the middle and 4.82 for the north, respectively). Another factor associated with depression among women was the number of comorbid chronic diseases (*P* for trend < 0.001). Among men, household income level and marital status were also found to be associated with the risk of depression. Specifically, the “Medium” household income appeared to be protective against depression (OR 0.15, 95% CI 0.03–0.68) for men, while the marital status of “Divorced/widowed/separated” was shown to be related to a dramatically higher risk compared to their peers who were “Married” (OR 3.50, 95% CI 1.35–9.12).

**Table 3:** The association between depression and socio-demographic factors and comorbid chronic diseases by gender (*N* = 8299)

	All			Men			Women		
	OR	95% CI		OR	95% CI		OR	95% CI	
Gender^a^									
Men	1.00								
Women	1.24	0.75	2.07						
Age group (years)^b^									
18–34	0.13	0.03	0.53	0.36	0.08	1.69			
35–44	0.22	0.09	0.54	0.23	0.05	1.06	0.22	0.07	0.64
45–54	0.30	0.14	0.65	0.41	0.13	1.35	0.24	0.09	0.66
55–64	0.60	0.33	1.10	0.73	0.28	1.90	0.54	0.25	1.16
≥ 65	1.00			1.00			1.00		
*P* for trend	< 0.001			0.025			< 0.001		
Residence^c^									
Urban	1.00			1.00			1.00		
Rural	2.24	1.33	3.78	2.39	1.05	5.45	2.17	1.10	4.28
Region^c^									
South	1.00			1.00			1.00		
Middle	3.13	1.45	6.79	2.06	0.67	6.33	4.47	1.48	13.51
North	3.57	1.69	7.55	2.60	0.91	7.42	4.82	1.62	14.30
*P* for trend	0.001			0.080			0.008		
Household income level ^c^									
Low	1.00			1.00			1.00		
Medium	0.31	0.14	0.68	0.15	0.03	0.68	0.46	0.18	1.16
High	0.60	0.32	1.15	0.58	0.22	1.53	0.61	0.26	1.47
*P* for trend	0.007			0.061			0.053		
Marital status^c^									
Married	1.00			1.00			1.00		
Single	1.01	0.13	7.59	1.41	0.17	11.34			
Divorced/widowed/separated	1.42	0.73	2.74	3.50	1.35	9.12	0.74	0.31	1.79
Number of comorbid NCDs^c,d^									
0	1.00			1.00			1.00		
1	1.41	0.54	1.85	0.78	0.32	1.92	1.20	0.51	2.82
2	2.07	0.95	4.51	0.66	0.14	3.16	3.71	1.42	9.72
≥ 3	3.83	1.33	11.02	3.36	0.66	17.04	4.29	1.06	17.35
*P* for trend	< 0.001			0.284			< 0.001		

^a^Adjusted for age (continuous)

^b^Adjusted for gender

^c^Adjusted for age (continuous) and gender

^d^Non-communicable chronic diseases, including cancer, stroke, myocardial infarction, hypertension, diabetes, chronic obstructive pulmonary disease and asthma

### Impacts on daily life functioning

**Fig. 1:**
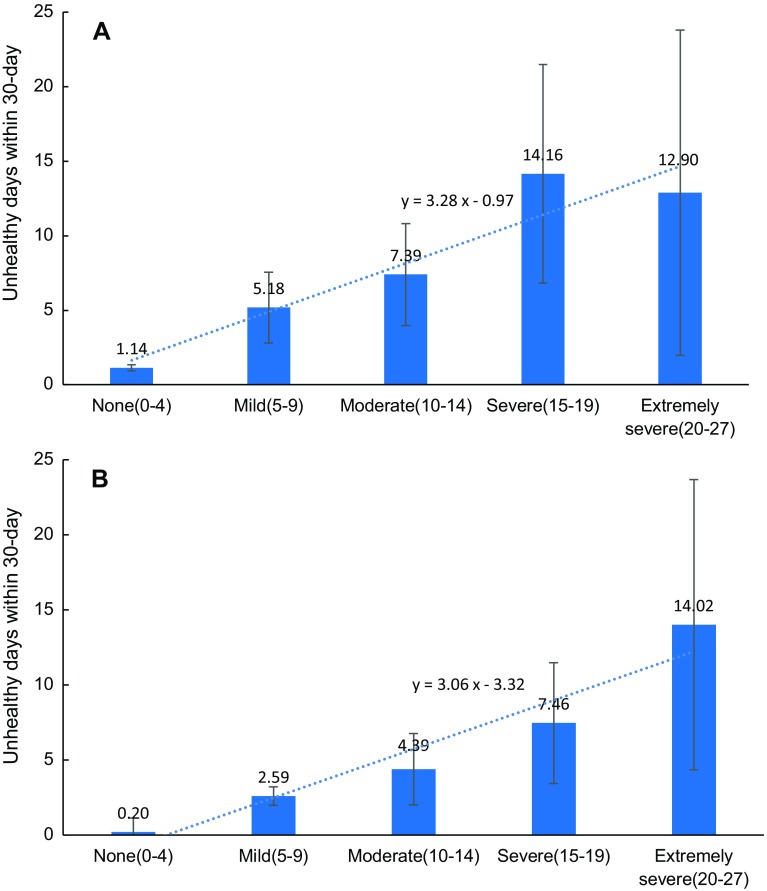
Unhealthy days within 30 days by the severity of depression: **a** caused by physical illnesses; **b** caused by mood problems. *Error bar for 95% confidence interval

Participants with depression reported an average of 11.75 unhealthy days caused by physical illnesses and 8.31 unhealthy days caused by mood problems within the previous 30 days, while these numbers were just 1.35 days and 0.32 days, respectively, among the non-depressed group (both *P* < 0.001). Data also suggested that the severity of depression was correlated with unhealthy days caused by physical illnesses (*r*_spearman_ = 0.24, *P* < 0.01) or mood problems (*r*_spearman_ = 0.29, *P* < 0.01). As shown in Fig. 1, the reported unhealthy days caused by both physical illnesses and mood problems within the previous 30 days were positively correlated to the severity of depression (both *P* for trend < 0.01). People with “severe” or “extremely severe” depression were bothered by physical illnesses in about one-third of the past 30 days. Unhealthy days caused by mood problems increased with the severity of depression, and people in the “extremely severe” group were affected by mood problems in nearly half of the previous 30 days (14.02 days).

**Fig. 2:**
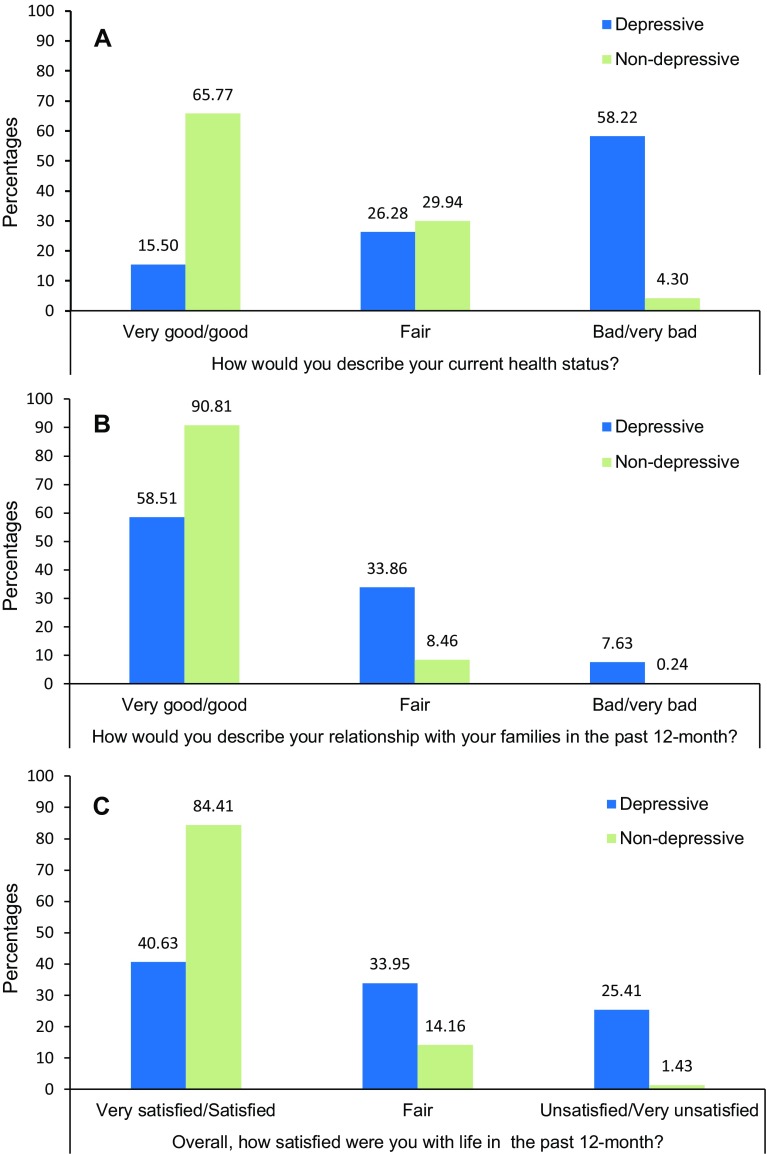
Comparison between depressive and non-depressive participants of: **a** self-rated health status; **b** relationships with families, **c** self-satisfaction with life

As demonstrated in Fig. 2, people with depression were also more likely to rate their health status as “Bad/Very bad” compared with non-depressed respondents (58.22 vs. 4.30%). Similarly, depressive participants were more likely to report a “Bad/Very bad” relationships with families (7.63 vs. 0.24%) or being “Unsatisfied/Very unsatisfied” with life in the past 12 months (25.41 vs. 1.43%). By contrast, only 15.50% of individuals with depression ranked their health status as “Good/Very good”, 58.51% ranked their relationships with families as “Good/Very good” and 40.63% ranked their satisfaction with life as “Satisfied/Very satisfied”, whereas the numbers in the non-depressive group were 65.77, 90.81 and 84.41%, respectively.

## Discussion

This study was conducted to assess the prevalence of depression and to investigate its associated factors and impacts on life functioning among the general adult population in the Jiangsu Province of China. Findings from this study contribute to the epidemiological knowledge of depression, thereby providing evidence for intervention development and health-care service delivery to adequately address this problem.

To the best of our knowledge, this is the first study utilising the PHQ-9 to screen for depression among a representative sample of the general adult population in a large area of China. The prevalence estimates of depression and major depression in this study (0.56 and 0.22%, respectively) are lower than most previous findings of China [12]. Zhou et al. [23] reported that the 2-week prevalence of depressive symptoms measured by the PHQ-9 in a rural Chinese population (aged ≥ 35) was 5.9%, while Lee et al. [29] found that the 1-year prevalence estimate of depression by CIDI in metropolitan China was 1.8%. The prevalence estimates of this study were also lower than the findings of Yu et al. [30], who reported a prevalence of 1.7% of major depressive disorder assessed by PHQ-9 in a Hong Kong population.

The exact reasons for the low estimates are not clear, but the use of the PHQ-9 might contribute to this. Only a small number of studies [22, 30–32] have tested the reliability and validity of the PHQ-9 in China and participants of these studies were some important subgroups of the population (e.g. elderly people, urban residents, clinical patients). However, validation studies among the general population are quite limited. Additionally, no consistency has been achieved on the cutoff point for depression among the Chinese. For example, Wang et al. [22] recommended a score of 7 as the cutoff point for metropolitan residents in China, but Wang et al. [32] suggested a score of 15 for older Chinese, while internationally a cut point of 10 is commonly used [15]. Therefore, more scientifically designed studies with more representative study population are warranted to evaluate the validity of the PHQ-9, especially the cutoff standards. Meanwhile, given the low prevalence estimates of depression by the PHQ-9 we have found in this study, together with a recent study which suggested that the use of the PHQ-9 may lead to a lower prevalence estimate of major depression than the CIDI [33], further studies examining the comparability of the PHQ-9 against other diagnostic instruments are also needed [12, 30]. These studies will help to enhance the utility of the PHQ-9 as a practical and affordable tool to be used in screening for depression.

Other reasons for the low prevalence estimates may include: ① individuals with depressive symptoms being more likely to be excluded for being selected due to their “possible un-cooperativeness” during an interview; ② the exclusion of those respondents with options of “Don’t know” or “Refuse to respond” to any of the items in the PHQ-9 may also contribute to the underestimation, as this group of people were more likely to have depression according to the findings of this study; ③ even though multiple strategies were employed (e.g. interviewers were strictly trained, information confidentiality was promised and private interview rooms were provided), we should still be aware of possible information bias, in other words, the possibility of intentionally under-reporting of depressive symptoms by participants because of stigmatising attitudes to mental health problems [34].

Several socio-demographic factors were found to be associated with the presence of depression, with the risk of depression among rural residents being 2.24 (95% CI 1.33–3.78) times higher than that of their urban counterparts after adjustment for potential confounders. The middle and north regions, which have a relatively high percentage of rural residents, were also found to have a dramatically higher risk of depression than the south region (ORs were 3.57 for the north and 3.17 for the middle). From a geographic disparity study, Pan et al. [35] also found that the prevalence of depressive symptoms was almost four times higher in participants from the north than those from the south. More than half of the Chinese population lives in rural areas, and poverty, engagement in heavy physical labour and inadequate investment in health care are commonly found in this population. In the past years, the increase of mental disorders in metropolitan China related to rapid socioeconomic changes has alarmed researchers and policy makers [29, 36], but the mental health of rural residents has not been sufficiently addressed. This finding calls for further research into the link between depression and access to mental health services in rural areas. Additionally, income level and being “Divorced/widowed/separate” significantly influenced the risk of depression among men, but not women. This may partly be explained by the fact that men bear more financial responsibility in Chinese families and men may derive relatively more benefits from marriage than women [37]. Such gender difference in associated factors of depression provides evidence for future targeted intervention.

Another prominent factor associated with depression in this sample was the comorbidity of depression and chronic diseases. Data showed that the risk of depression increased with the number of comorbid chronic diseases, and respondents with three or more comorbidities were 3.83 times (95% CI 1.33–11.02) more likely to have depression than those without chronic disease, which is consistent with the findings of Chin et al. [31] from a prospective cohort study. The association of mental disorders and chronic disease has been well established [38], which could be partly explained by their common behavioural risk factors or neuroendocrine responses [10, 11]. Given the already prevalent and still increasing prevalence of chronic diseases in China [39, 40], the problem of depression among chronic disease patients should be addressed in clinical settings, as well as in health plans and resource allocation.

We found that depression, together with its severity, was associated with the number of unhealthy days. Cross-cultural studies have suggested that the experience of depression is more likely to be somesthetic (i.e. symptoms of pain, dizziness, fatigue, etc.) rather than psychological among the Chinese people [41], which may partially explain the greater number of unhealthy days reported by individuals with depression. Data also showed that depression was associated with low self-rated health status, poor relationships with families and low self-satisfaction with life. While it is hard to judge by this cross-sectional study whether the presence of depression leads to poorer life functioning or vice versa or whether they mutually interact, these findings imply the association of depression and poorer life functioning at a population level, with profound consequences for a country with a population size of China. This study provides further support for the development of an efficient mental health-care system in China, particularly that which can improve services to under-served populations.

## Strengths and limitations

There are several strengths of this study. First, a multi-stage stratified cluster random sampling scheme helped to recruit a representative sample of the general population within a large geographical area in China. Second, comprehensive information, covering demography, socioeconomic, chronic disease and life performance, was collected, enabling a sophisticated association analysis of depression in a single study. Furthermore, the survey procedure involved quality control measures, such as systematic training of the survey teams, regular supervision and random data quality checks, which were implemented to ensure data quality.

Nevertheless, it is important to note the limitations of the study: Firstly, no other depression-measuring instruments were used in parallel in this study, which limits our understanding of the reliability of prevalence estimates. Secondly, considering the fact that some study participants are difficult to read by themselves (due to limited literacy among rural residents, poor vision among elderly people, etc.), a face-to-face interview was used to collect data rather than a self-report method which is commonly used in other societies [20, 33]. The rationality of collecting information in a face-to-face way, especially when it concerns sensitive health issues like mental disorders, needs further reflection. Thirdly, the study sample was similar to the provincial population in most socio-demographic characteristics; however, due to the community-based sampling scheme used, fewer young people (who were mostly at universities or in workplaces, rather than in households) were recruited. Although the data were weighted according to the census population, this may still influence the reliability of the prevalence estimates. Despite the above limitations, this study enriches the epidemiological evidence of depression and its associated factors among the general population in China, and it also provides useful information for targeted population interventions and health-care service provision.

## Conclusions

A low prevalence of depression was found in this population of China, though the extent to which it reflects issues related to the measurement and/or other factors of the survey is not clear. Depression was found to be related to poorer health and poorer daily life functioning. Further research into the link between depression and access to services in rural areas is necessary. Meanwhile, depression among chronic disease patients should be addressed in clinical settings, health plans and resource allocation.
